# Competitive Anxiety as a Predictor of the Occurrence, Quantity, and Severity of Injuries in Young Cuban Athletes

**DOI:** 10.3390/ijerph23030354

**Published:** 2026-03-11

**Authors:** Jesús Ríos-Garit, Yanet Pérez-Surita, Verónica Gómez-Espejo, Mario Reyes-Bossio, Verónica Tutte-Vallarino

**Affiliations:** 1 Centro de Estudio de Actividad Física y Deporte, Universidad Central “Marta Abreu” de Las Villas, Santa Clara 50100, Cuba; 2 Facultad de Cultura Física, Universidad Central “Marta Abreu” de Las Villas, Santa Clara 50100, Cuba; yanetsurita@uclv.cu; 3 Facultad de Psicología y Logopedia, Universidad de Murcia, 30100 Murcia, Spain; veronica.gomez2@um.es; 4 Facultad de Psicología, Universidad Peruana de Ciencias Aplicadas, Lima 15001, Peru; mario.reyes@upc.pe; 5 Departamento de Bienestar y Salud, Universidad Católica del Uruguay, Montevideo 11600, Uruguay; vtutte@ucu.edu.uy

**Keywords:** competitive anxiety, young athletes, sports injuries

## Abstract

**Highlights:**

**Public health relevance—How does this work relate to a public health issue?**

- Sports injuries in young people are a growing problem that affects physical health, psychological well-being, and continuity in sports.
- Competitive anxiety emerges as a psychological risk factor that influences the occurrence, number, and severity of injuries.

**Public health significance—Why is this work of significance to public health?**

- It connects physical health and mental health, showing that emotional states directly influence musculoskeletal integrity.
- It reinforces the need to incorporate psychological assessment into prevention programs, optimizing resources and reducing healthcare costs.

**Public health implications—What are the key implications or messages for practitioners, policy makers and/or researchers in public health?**

- Competitive anxiety can be used as an early indicator of risk, allowing for timely interventions before injuries occur.
- Sports policies should promote systematic psychological monitoring protocols during training cycles.

**Abstract:**

Previous studies suggest that elevated competitive anxiety may increase the likelihood of injury. The present research aims to examine the role of competitive anxiety as a predictor of injury occurrence, frequency, and severity. A cross-sectional, correlational design was conducted with 131 athletes (mean age = 16.49 years), predominantly male. Injury data were obtained through medical record review, and competitive anxiety was assessed using the Competitive Anxiety Inventory-2. Empirical frequency distributions, descriptive statistics, non-parametric tests, and logistic and ordinal regression models were employed. A high incidence of injuries was observed, although most were minor. Competitive anxiety was characterized by elevated levels of cognitive anxiety and self-confidence. Injured athletes exhibited greater overall competitive anxiety (r = 0.31, *p* < 0.001), with higher levels observed among those who sustained more injuries (ε^2^ = 0.12, *p* = 0.001), and a very large effect was found in relation to injury severity (ε^2^ = 0.17, *p* < 0.001). The occurrence of injury can only be predicted in 10.9–14.7% of cases through increased cognitive and somatic anxiety, whereas an increase across all dimensions of competitive anxiety predicts a greater number (13–14%) and severity (20.3–21.8%) of injuries. These findings underscore the importance of developing skills to manage competitive anxiety, particularly its cognitive dimension and maintaining optimal levels of self-confidence in young athletes.

## 1. Introduction

Research on the etiology of sports injuries has advanced considerably in recent decades, with psychological factors now recognized as critical determinants of injury risk. Among these, competitive anxiety has been consistently identified as a central construct, exerting influence through disruptions in attentional focus, decision-making, and motor coordination, thereby heightening susceptibility to technical errors and subsequent injury. The foundational model of Andersen and Williams [1] remains influential in framing this interaction, emphasizing the interplay between situational stressors and coping resources.

Yet, more recent empirical evidence has shown that elevated anxiety levels are associated with a greater likelihood of sustaining sport injuries across different playing positions and competitive contexts [2]. Subsequent studies further refined this perspective by identifying specific risk profiles in which competitive anxiety and dispositional competitiveness interact to increase injury vulnerability [3]. Additional research in football has shown that anxiety coexists with other psychological characteristics, such as resilience, and varies according to competitive level, reinforcing its role as a determinant of injury risk [4]. More recently, longitudinal analyses in high-performance environments have confirmed that fluctuations in competitive anxiety and mood states are closely linked to injury occurrence, underscoring the dynamic nature of this psychological–physical interaction [5].

From this perspective, contemporary research has expanded on these postulates, confirming that psychological factors not only influence the incidence of injuries but also the rehabilitation processes and performance after the injury occurs [6]. In soccer players, for example, it has been documented that injury induces alterations in pre-competitive anxiety and mood, affecting return to sport [7]. Subsequent research has consolidated the notion that effective prevention must necessarily incorporate psychological variables alongside traditional physical and biomechanical ones [8]. More recently, it has been argued that the risk of injury is not only associated with stress, but also with a specific profile of negative emotional states that includes high anxiety, depressive symptoms, and low self-confidence, which increases the athlete’s vulnerability [9].

This evidence positions psychological factors as essential components in explanatory models of sports injury. Specifically, competitive anxiety has been associated with an increased risk of musculoskeletal injuries, mediated by its negative impact on concentration, emotional regulation, and muscle tension [1]. This association is particularly relevant in team sports. A recent systematic review focusing on basketball, handball, and volleyball [10] not only confirms the high injury burden in these sports but also explicitly identifies the need for in-depth research into psychosocial risk factors, beyond purely physical and technical ones. This call from the literature reinforces the relevance of studying variables such as competitive anxiety in these specific contexts.

In youth populations, recent findings lend relevance to this association. Sánchez-Ruiz et al. demonstrated in young soccer players that high levels of competitive anxiety predicted a higher incidence of injuries, a relationship that was moderated by the competitive category, being more intense at higher levels of demand [11]. This study suggests that anxiety not only increases the likelihood of injury but may also be linked to its severity and frequency, depending on the sporting context. However, its exclusive focus on soccer highlights a critical gap pointed out by Milić et al. [10]: the lack of longitudinal and comprehensive research on psychological predictors in other team sports with similar dynamics, where competitive pressure and injury risk are equally high.

Convergently, a recent meta-analysis corroborates the bidirectional nature of this relationship, establishing that anxiety predisposes to injury and, in turn, the experience of injury exacerbates anxiety symptoms, creating a potentially harmful cycle for the athlete’s health [12]. However, not all research has found confirmatory evidence of the relationship between the two variables. Several studies have not found significant relationships between competitive anxiety and injuries [13,14], while it was recently determined that this relationship may be conditioned by other factors, since although significant relationships between injury and somatic anxiety were found in the total sample, when the analysis was segmented, these relationships were not found in younger athletes [11].

In the specific context of Cuba, research on sports injuries has shifted from epidemiological approaches to an incipient integration of the psychological dimension. Longitudinal studies such as that by Martínez-Estupiñán on child athletes, which reported a high incidence of injuries, already pointed to the need to incorporate psychosocial factors into prevention programs [15]. Continuing along these lines, Ríos Garit and Pérez Surita identified that both competitive anxiety and extreme levels of self-confidence were associated with a higher incidence of injuries in high-performance basketball players, providing local empirical evidence on this interaction [16]. Recently, evidence has also been obtained on the relevance of competitive anxiety as a significant predictor of injuries when interacting with psychological skills in Cuban athletes from various team sports [17].

Despite these advances, inconsistencies in the accumulated evidence persist. The synthesis by Milić et al. [10] highlights that, although the epidemiology of injuries in sports such as basketball, handball, and volleyball is well known, there is a notable lack of studies designed to examine causal relationships, particularly with psychological variables in these populations. Therefore, the main objective of this study is to determine the role of competitive anxiety as a predictor of the occurrence, number, and severity of injuries in young Cuban athletes in team sports, responding to the call for recent research to delve deeper into psychosocial predictors in these sports contexts.

## 2. Materials and Methods

### 2.1. Study Design

A cross-sectional and correlational study was conducted on volleyball, basketball, baseball, and soccer teams in the province of Villa Clara at the end of the Special Preparation stage of the 2024–2025 training macrocycle (between the first week of May and the last week of June 2025). The aim was to analyze the population of young athletes participating in team sports in the province of Villa Clara who were in the preparation stage for the main competition of 2025.

### 2.2. Participants

A total of 131 athletes aged between 12 and 23 years (M = 16.49) and with between 1 and 13 years of practice (M = 7.08) were studied. Males predominated with 79.4% (*n* = 104). The least represented sport was volleyball and the under-23 category in general, as shown in Table 1. A non-probabilistic sample was taken based on the following criteria:

Inclusion
  - Young athletes in team sports who are training for the 2025 championship.

Exclusion
  - Athletes who did not give their consent to participate.
  - Athletes whose parents or guardians do not approve of their participation in the study.

**Table 1:** Distribution of the sample in relation to sport and category.

Variables		N	%
Sport	Baseball	45	34.4
	Basketball	26	19.8
	Soccer	47	35.9
	Volleyball	13	9.9
Category	U-15	47	35.9
	U-18	46	35.1
	U-23	38	29.0

### 2.3. Instruments

Data on injuries were obtained by reviewing the medical records of athletes at the Provincial Sports Medicine Center in Villa Clara. The variables injury (Uninjured, Injured); number of injuries (1 injury, 2 injuries, more than 2 injuries) and severity of injury (Mild, Moderate, Severe, Very Severe) are classified as qualitative. The classification of injuries by severity was carried out following the criteria proposed by Olmedilla et al. [18] (minor injuries require treatment without interrupting training; moderate injuries require treatment and necessitate missing at least one day of training or competition; severe injuries involve one or more months of sports inactivity, often requiring hospitalization and/or surgical intervention; and very severe injuries lead to a permanent decrease in athletic performance, requiring ongoing rehabilitation to prevent further deterioration).

Competitive Sport Anxiety Inventory, Spanish version [19]: this instrument was used to assess competitive anxiety. It consists of 27 items distributed across three subscales that measure cognitive anxiety, somatic anxiety, and self-confidence with four Likert-type response options (1 = Not at all; 2 = A little; 3 = Moderately; 4 = Very much). The instrument showed adequate reliability indices in the present study, both for the subscales (α = 0.82 for Cognitive Anxiety; α = 0.81 for Somatic Anxiety; α = 0.76 for Self-Confidence), as well as for the total variable (α = 0.85 for Competitive Anxiety).

### 2.4. Procedures

Competitive anxiety was assessed at the beginning of the special preparation phase in all selected teams. Subsequently, at the end of the phase, access to the injury record was obtained. The summary of injury data was provided by each sports medicine specialist after reviewing the medical records. The CSAI-2 instrument was administered in printed form, at each sport’s venue, in agreement with the head coach, and in the morning. In each sport, it was administered by the corresponding sports psychology specialist. Before beginning the tests, their characteristics, how to complete them, and the objectives of the study were explained in detail, with an emphasis on the accuracy of the information.

### 2.5. Ethical Considerations

The study is part of the project “Tools for the biopsychosocial prevention of injuries in team sports,” which is associated with the Sectoral Program “Sport and Human Development” of the National Institute of Sport, Physical Education, and Recreation of Cuba. The research was approved by the Scientific Council of the Provincial Center for Sports Medicine of Villa Clara and the Medical Research Ethics Committee (0101/2025). Its development strictly adhered to the ethical precepts of scientific research contained in the Declaration of Helsinki [20].

### 2.6. Data Analysis

Empirical frequency distribution was used to describe the distribution of cases in terms of the presence of injury, number of injuries, and severity of injuries. Descriptive statistics such as mean, deviation, asymmetry, and kurtosis were used for the variables of age, sporting experience, and competitive anxiety with their dimensions. The Kolmogorov–Smirnov test for normality of data for a sample indicated a non-normal distribution. Consequently, the Mann–Whitney U test was used to compare the variables of age, sporting experience, and competitive anxiety among athletes according to the presence or absence of injuries. The Kruskal–Wallis test was used to compare these same variables in relation to the number and severity of injuries suffered. The effect size was calculated for Mann–Whitney U using the r coefficient, obtained from the standardized Z statistic (r = Z/√N), following the recommendations of Fritz et al. [21]. For multiple group comparisons with Kruskal–Wallis, the effect size was calculated using epsilon squared (ε^2^ = H/N − 1) according to Tomczak and Tomczak [22]. A binary logistic regression model was used to predict the injury variable, and ordinal regression models were used for the variables of number and severity of injuries. The SPSS version 25.0 software package for Windows was used, considering a 95% confidence interval.

## 3. Results

**Figure 1:**
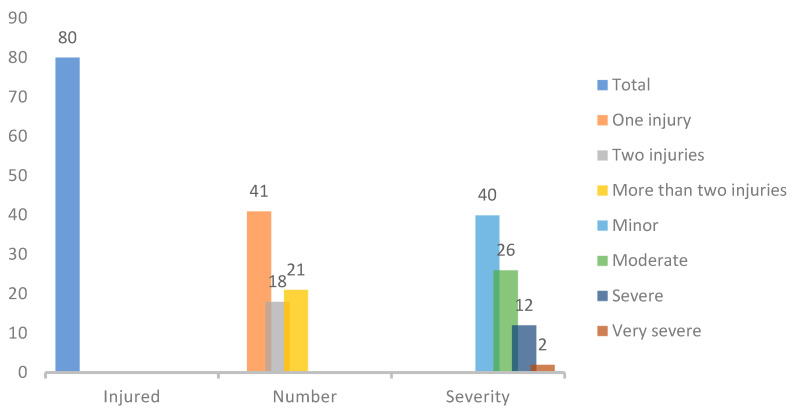
Characteristics of injuries.

Figure 1 describes the injuries during the 2024–2025 training macrocycle. There is a high occurrence and notable number of injuries, although those of lesser severity predominate.

Table 2 shows the values of the descriptive statistics and the normal distribution test of the data. Only the competitive anxiety variable has a normal distribution. Self-confidence and cognitive anxiety exhibit high values.

Table 3 describes the results of the comparison of chronological age, sporting experience expressed in years of practice, and competitive anxiety. Only self-confidence does not differ significantly between injured and non-injured athletes. Injured athletes have higher competitive anxiety with a moderate effect size (r = 0.31), which is configured through higher cognitive and somatic anxiety with small individual effects (r = 0.27 and r = 0.24, respectively).

Table 4 shows that the number of injuries suffered significantly differentiates competitive anxiety as a general variable, but at the expense of cognitive and somatic anxiety, in that order. A large effect is obtained in general competitive anxiety (ε^2^ = 0.12), cognitive anxiety (ε^2^ = 0.08), and somatic anxiety (ε^2^ = 0.06), as well as a moderate effect in self-confidence (ε^2^ = 0.02), although not significant.

Table 5 analyzes the differences in variables in relation to the severity of injuries. It can be seen that competitive anxiety as a general variable differs significantly depending on the severity of injuries, with a very large effect size (ε^2^ = 0.17). In this regard, cognitive anxiety (ε^2^ = 0.12) and somatic anxiety (ε^2^ = 0.08) differ significantly with a large effect in both cases.

Table 6 shows the logistic regression data for predicting injury. It can be seen that cognitive and somatic anxiety are significant predictors of injury, but self-confidence is not. For each point increase in cognitive anxiety, the probability of injury increases by 9.7%, while somatic anxiety increases by 11%. The model demonstrated an acceptable global fit (−2 Log Likelihood = 160.059; X^2^ = 15.072, *p* = 0.002). The Hosmer–Lemeshow test was not significant (X^2^= 6.195, *p* = 0.625). In terms of predictive performance, the model correctly classified 68.7% of the cases, with a sensitivity of 87.5% for injured athletes and a specificity of 39.2% for non-injured athletes. These results suggest a moderate discriminative ability, with stronger effectiveness in identifying positive cases.

Table 7 shows the results of the ordinal regression to predict the number and severity of injuries. With regard to the number of injuries, the three dimensions of competitive anxiety are significant predictors. However, the variable that best predicts the number of injuries is cognitive anxiety, followed by somatic anxiety and self-confidence, all in a positive regard. With regard to the severity of injuries, the results follow the same logic; however, the data show that the three dimensions of competitive anxiety predict severity to a greater extent than the number of injuries (Severity = 20.3–21.8%, Number = 13–14%). The model for the number of injuries demonstrated a good overall fit (−2 Log likelihood = 318.815; X^2^ = 18.232, df = 3, *p* = < 0.001). Goodness of fit statistics were satisfactory (Pearson X^2^ = 409.270, *p* = 0.129; Deviance X^2^ = 316.042, *p* = 0.991). The test of parallel lines was not significant (X^2^ = 6.025, df = 6, *p* = 0.420), supporting the proportional odds assumption. Similarly, the model for injury severity showed an adequate fit (−2 Log likelihood = 316.822; X^2^ = 29.719, df = 3, *p* = < 0.001). Goodness-of-fit indices were excellent (Pearson X^2^ = 542.934, *p* = 0.118; Deviance X^2^ = 314.049, *p* = 1.000) and the proportional odds assumption was met (X^2^ = 1.953, df = 9, *p* = 0.992). Together, these results suggest that both models provide a moderate discriminative ability across categories of injury frequency and severity.

## 4. Discussion

It was found that most athletes had suffered minor injuries at least once during the preparation period analyzed. These data confirm the negative epidemiological behavior of injuries from an early age in team sports [10,15]. This finding in young athletes coincides with the results reported by a previous study in a sample of older high-performance athletes in the same context [23]. The contrasting evidence highlights the importance and necessity of a sports injury care system focused on timely prevention.

With regard to the relationship between competitive anxiety and injuries in these young athletes, it was found that competitive anxiety reaches significantly higher levels in those who have been injured more frequently and, especially, in those who have suffered more severe injuries. This finding reinforces the concept of sports injuries as a multifactorial phenomenon in which psychological factors play a relevant role [1,24,25].

On the other hand, competitive anxiety showed significant differences between injured and non-injured athletes, which agrees with the results shown in the meta-analysis by Chow et al. [12], where a significant relationship is established between mental health variables and sports injuries in adolescents. This suggests that anxiety is a relevant factor within a more complex process that includes physical demands, workload, and contextual factors.

A more specific analysis showed that cognitive anxiety was the dimension with the greatest predictive weight, both in terms of the number and severity of injuries. In this regard, excessive worry, anticipation, or intrusive thoughts can compromise concentration and increase the likelihood of errors. Likewise, somatic anxiety showed significant predictive power, both in the occurrence and in the number and severity of injuries. These findings are consistent with other studies that argue that negative emotional states are associated with physiological alterations that can affect coordination and increase the occurrence of injuries [9,26].

Competitive anxiety explained a greater percentage of variance in injury severity than in the mere occurrence of injury. This result suggests that anxiety not only increases the likelihood of injury but may also be linked to mechanisms that aggravate the damage. Along the same lines, previous studies [9,11] suggest that negative emotional profiles can intensify the physiological response to stress and limit the ability to adapt to risky situations. In this regard, a study of young soccer players found that the physiological and cognitive changes caused by high levels of competitive anxiety increased the risk of injury [27].

In this regard, competitive anxiety may also be interpreted within a broader framework of psychological demands in sport. Although variables such as mental workload [28] or emotional exhaustion [29] were not directly assessed in this study, the previous literature suggests that these factors may interact with anxiety, amplifying cognitive overload and reducing attentional efficiency. This interaction could partially explain why anxiety shows a stronger association with injury severity than with mere occurrence, as higher psychological demands may impair both perception and response to risk situations.

With regard to self-confidence, although no significant association was found with the occurrence of injury, its predictive capacity was examined in terms of the quantity and severity. This suggests that self-confidence may not influence whether an athlete gets injured, but rather how they behave once exposed to risk situations. Excessively high levels of self-confidence could lead to risk-taking behaviors, underestimation of danger, or delayed recognition of physical warning signals, contributing to more frequent or more severe injuries. This interpretation highlights the need to consider not only deficits but also maladaptive excesses in psychological variables.

This result is similar to that found in the Cuban population by Ríos Garit and Pérez Surita [16], who identified that extreme levels of self-confidence may be associated with a higher incidence of injury. In this regard, very high self-confidence can be a risk factor, which would increase the probability of recurrence or aggravation of the injury. This leads to the need for interventions focused on developing skills for the proper management of anxiety in competition and maintaining optimal levels of self-confidence in these young Cuban athletes, but more markedly in those who have been injured more often and have suffered more severe injuries.

From an applied perspective, the results support the need to implement psychological intervention programs aimed at the proper management of competitive anxiety and the maintenance of optimal levels of self-confidence. In line with other studies, affective injury prevention should incorporate mental health aspects into sports preparation plans [8,17,30]. Furthermore, the evidence obtained suggests that competitive anxiety is a significant predictor of the number and severity of injuries in these young athletes, reinforcing the need to adopt a biopsychosocial approach to sports injury prevention in Cuba, integrating the assessment and training of psychological variables into the athlete’s preparation process [17].

This study presents several limitations that should be considered when interpreting the findings. First, the cross-sectional and correlational design does not allow for definitive causal inferences between competitive anxiety and the occurrence of injuries. Although anxiety was assessed at the beginning of the preparation phase and injuries were recorded afterward, the possibility of reverse causality cannot be ruled out—that is, previous injury experiences may have increased the levels of competitive anxiety observed in athletes. The literature highlights the bidirectional nature of this relationship, suggesting that future research should employ longitudinal designs or cross-lagged regression models to examine how both variables influence each other over time.

Second, the sample size and its non-probabilistic nature limit the generalizability of the results to other populations of young athletes, both within and outside the Cuban context. The predominance of male athletes and the underrepresentation of certain sports, such as volleyball, also restrict the extrapolation of findings to disciplines with different competitive dynamics or to populations with greater gender balance.

Third, the study focused exclusively on psychological variables and medical records of injuries, without incorporating other relevant factors such as training load, environmental conditions, social support, or individual coping strategies. These factors may interact with competitive anxiety in explaining the occurrence and severity of injuries.

Finally, although validated instruments were used to measure competitive anxiety, the self-reported nature of these questionnaires may be subject to response bias, particularly in young populations who may face difficulties in accurately recognizing or expressing their emotional states.

Building on the limitations identified, several avenues for future research are suggested. First, longitudinal and experimental designs should be employed to better establish the causal direction of the relationship between competitive anxiety and sports injuries, and to disentangle the potential bidirectional effects. Incorporating cross-lagged panel models would allow researchers to examine how anxiety and injury mutually reinforce each other over time.

Second, future studies should expand the sample size and adopt probabilistic sampling methods to enhance the generalizability of findings. Greater representation of female athletes and underrepresented sports, such as volleyball, would provide a more comprehensive understanding of how competitive anxiety operates across different contexts and populations.

Third, research should integrate additional explanatory variables beyond psychological measures and medical records in Cuban athletes. Factors such as training load, environmental conditions, social support, and coping strategies may interact with competitive anxiety and contribute to injury risk. Multidimensional models that combine psychological, physical, and social predictors would provide a more holistic view of injury etiology.

Finally, future work should consider the limitations of self-reported measures of anxiety. Complementing questionnaires with physiological indicators (e.g., heart rate variability, cortisol levels) or behavioral observations could reduce response bias and improve the accuracy of psychological assessments in young athletes.

## 5. Conclusions

Competitive anxiety reached higher levels in young athletes who have been injured more frequently, but especially in those who have suffered more severe injuries. Not all dimensions of competitive anxiety have the same degree of influence on the occurrence of injury. However, an increase in the number and severity of injuries can be predicted when all dimensions of competitive anxiety increase. Although the regression models indicate that competitive anxiety is a significant predictor of injury-related variables, the proportion of explained variance remains moderate. This suggests that anxiety should be considered as one contributing factor within a multifactorial model, where physical, contextual, and other psychological variables also play a relevant role. Therefore, the predictive capacity of anxiety should be interpreted with caution, avoiding deterministic conclusions. Despite these limitations, the findings suggest that very highly competitive anxiety can be a risk factor, which raises the need for interventions focused on developing skills for the proper management of anxiety in competition and maintaining optimal levels of self-confidence in these young Cuban athletes, but more so in those who have been injured more often and have suffered more severe injuries.

## Figures and Tables

**Table 2:** Description of the variables and data normality analysis.

Variables	Mean	SD	Skewness	Kurtosis	K-S	*p*
Chronological age	16.49	2.915	0.651	−0.005	0.138	<0.001
Sports experience	7.08	4.108	0.340	−0.951	0.117	<0.001
Cognitive anxiety	30.69	6.161	1.111	5.760	0.118	<0.001
Somatic anxiety	20.71	5.237	0.727	0.741	0.090	0.012
Self-confidence	36.60	6.912	−0.922	0.243	0.137	<0.001
Competitive anxiety	88.00	9.459	0.045	2.783	0.072	0.092

Note. K-S = Kolmogorov–Smirnov.

**Table 3:** Comparison of variables related to the occurrence of injury.

Variables	Factor	N	Average Range	U	Z	*p*	r
Chronological age	Not injured	51	64.62	1969.500	0.335	0.738	0.02
	Injured	80	66.88				
Sports experience	Not injured	51	64.80	1979.000	0.289	0.773	0.02
	Injured	80	66.76				
Cognitive anxiety	Not injured	51	53.21	1387.500 **	3.088	0.002	0.27
	Injured	80	74.16				
Somatic anxiety	Not injured	51	54.35	1446.600 **	2.810	0.005	0.24
	Injured	80	73.43				
Self-confidence	Not injured	51	65.14	1996.000	0.208	0.835	0.01
	Injured	80	66.55				
Competitive anxiety	Not injured	51	51.25	1287.500 ***	3.556	<0.001	0.31
	Injured	80	75.41				

Note. ** *p* < 0.01; *** *p* < 0.001 (two-tailed significance); r = 0.10 (small effect); r = 0.30 (moderate effect); r = 0.50 (large effect).

**Table 4:** Comparison of variables in relation to the number of injuries suffered.

Variables	Factor	N	Average Range	H	*p*	ε^2^
Chronological age	None	51	64.62	0.201	0.977	0.00
	One injury	41	67.39			
	Two injuries	18	64.58			
	More than two injuries	21	67.86			
Sports experience	None	51	64.80	0.907	0.824	0.00
	One injury	41	65.73			
	Two injuries	18	73.56			
	More than two injuries	21	62.95			
Cognitive anxiety	None	51	53.21	10.973 *	0.012	0.08
	One injury	41	71.38			
	Two injuries	18	70.58			
	More than two injuries	21	82.64			
Somatic anxiety	None	51	54.35	8.517 *	0.036	0.06
	One injury	41	76.54			
	Two injuries	18	68.64			
	More than two injuries	21	71.45			
Self-confidence	None	51	65.14	3.337	0.343	0.02
	One injury	41	59.48			
	Two injuries	18	70.06			
	More than two injuries	21	77.36			
Competitive anxiety	None	51	51.25	16.291 **	0.001	0.12
	One injury	41	68.89			
	Two injuries	18	75.19			
	More than two injuries	21	88.31			

Note. * *p* < 0.05; ** *p* < 0.01 (two-tailed significance); ε^2^ ≤ 0.01 (small effect); ε^2^ > 0.01–0.06 (moderate effect); ε^2^ > 0.06–0.14 (large effect); ε^2^ > 0.14 (very large effect).

**Table 5:** Comparison of variables in relation to the severity of injuries.

Variables	Factor	N	Average Range	H	*p*	ε^2^
Chronological age	None	51	64.62	2.989	0.560	0.02
	Minor	40	64.38			
	Moderate	26	64.94			
	Severe	12	82.67			
	Very severe	2	47.50			
Sports experience	None	51	64.80	4.547	0.337	0.03
	Minor	40	61.24			
	Moderate	26	71.75			
	Severe	12	80.13			
	Very severe	2	32.25			
Cognitive anxiety	None	51	53.21	15.838 **	0.003	0.12
	Minor	40	65.30			
	Moderate	26	77.46			
	Severe	12	90.96			
	Very severe	2	107.50			
Somatic anxiety	None	51	54.35	10.579 *	0.032	0.08
	Minor	40	72.11			
	Moderate	26	67.92			
	Severe	12	85.63			
	Very severe	2	98.00			
Self-confidence	None	51	65.14	5.895	0.207	0.04
	Minor	40	63.45			
	Moderate	26	62.69			
	Severe	12	89.50			
	Very severe	2	41.00			
Competitive anxiety	None	51	51.25	22.774 ***	<0.001	0.17
	Minor	40	67.00			
	Moderate	26	72.96			
	Severe	12	105.33			
	Very severe	2	95.75			

Note. * *p* < 0.05; ** *p* < 0.01; *** *p* < 0.001 (two-tailed significance); ε^2^ ≤ 0.01 (small effect); ε^2^ > 0.01–0.06 (moderate effect); ε^2^ > 0.06–0.14 (large effect); ε^2^ > 0.14 (very large effect).

**Table 6:** Logistic regression to predict the occurrence of injury through the dimensions of competitive anxiety.

Criterion	Pseudo R Squared		Interactions	B	SE	Wald	gl	*p*	Exp(B)	95% C.I. for EXP(B)
Injury	R-squared of Cox & Snell	0.109	Cognitive anxiety	0.092	0.037	6.228	1	0.013	1.097	1.020–1.179
			Somatic anxiety	0.104	0.042	6.238	1	0.013	1.110	1.023–1.204
	R-squared of Nagelkerke	0.147	Self-confidence	0.040	0.030	1.741	1	0.187	1.041	0.981–1.105
			Constant	−5.950	2.079	8.195	1	0.004	0.003	-

**Table 7:** Ordinal regression to predict the number and severity of injuries across the dimensions of competitive anxiety.

Models	Pseudo R Squared		Interactions		Estimate	Std. Error	Wald	gl	*p*	95% Confidence Interval
Injuries number	Cox & Snell	0.130	Threshold	One injury	6.151	1.765	12.148	1	<0.001	2.692–9.611
				Two injuries	7.596	1.806	17.688	1	<0.001	4.056–11.136
				>2 injuries	8.465	1.832	21.361	1	<0.001	4.875–12.055
	Nagelkerke	0.140	Location	Cognitive anxiety	0.092	0.030	9.306	1	0.002	0.033–0.151
				Somatic anxiety	0.083	0.035	5.777	1	0.016	0.015–0.151
				Self-confidence	0.057	0.027	4.618	1	0.032	0.005–0.109
Injuries Severity	Cox & Snell	0.203	Threshold	Minor	8.046	1.824	19.467	1	<0.001	4.472–11.620
				Moderate	9.514	1.871	25.861	1	<0.001	5.847–13.180
				Severe	11.024	1.928	32.686	1	<0.001	7.245–14.803
				Very severe	13.502	2.171	38.667	1	<0.001	9.246–17.757
	Nagelkerke	0.218	Location	Cognitive anxiety	0.134	0.030	19.610	1	<0.001	0.075–0.193
				Somatic anxiety	0.106	0.035	8.963	1	0.003	0.037–0.175
				Self-confidence	0.062	0.027	5.250	1	0.022	0.009–0.116

## Data Availability

The original contributions presented in this study are included in the article. Further inquiries can be directed to the corresponding author.

## References

[1] Andersen M.B., Williams J.M. (1988). A model of stress and athletic injury: Prediction and prevention. J. Sport Exerc. Psychol..

[2] Fernández R., Zurita-Ortega F., Linares D., Ambros J., Pradas F., Linares M.M. (2014). Relación entre la ansiedad estado/ rasgo, posición en el terreno de juego y ocurrencia de lesiones deportivas. Univ. Psychol..

[3] Prieto J.M., Labisa A., Olmedilla A. (2015). Ansiedad competitiva, competitividad y vulnerabilidad a la lesión deportiva: Perfiles de riesgo. Rev. Iber. Psicol. Ejerc. Deporte.

[4] Zurita-Ortega F., Rodríguez-Fernández S., Olmo-Extremera M., Castro-Sánchez M., Chacón-Cuberos R., Cepero-González M. (2017). Análisis de la resiliencia, ansiedad y lesión deportiva en fútbol según el nivel competitivo. Cult. Cienc. Deporte.

[5] Boladeras A., Gil-Caselles L., Moreno-Fernández I., Guillén-Cots J., Garcia-Naveira A., Ruiz-Barquín R., Olmedilla-Zafra A. (2025). The Relationship Between Mood, Competitive Anxiety, and Injuries: A Longitudinal Analysis in High-Performance Female Volleyball Players. Appl. Sci..

[6] Ríos-Garit J., Berengüí-Gil R., Solé-Cases S., Pérez-Surita Y., Cañizares-Hernández M., Cárdenas Rodríguez R. (2024). Ansiedad, estados de ánimo y habilidades psicológicas en jóvenes deportistas lesionados en proceso de rehabilitación. Rev. Psicol. Apl. Dep. Ejer. Físico.

[7] Olmedilla A., Ortega E., Gómez J.M. (2014). Influencia de la lesión deportiva en los cambios del estado de ánimo y de la ansiedad precompetitiva en futbolistas. Cuad. Psicol. Deporte.

[8] Gil-Caselles L., Olmedilla A. (2024). Links between sports injuries and mental health in elite athletes: The current state of affairs. Arch. Med. Deporte.

[9] Olmedilla A., Martins B., Ponseti-Verdaguer F.J., Ruiz-Barquín R., García-Mas A. (2022). It Is Not Just Stress: A Bayesian Approach to the Shape of the Negative Psychological Features Associated with Sport Injuries. Healthcare.

[10] Milić V., Radenković O., Čaprić I., Mekić R., Trajković N., Špirtović O., Koničanin A., Bratić M., Mujanović R., Preljević A. (2025). Sports Injuries in Basketball, Handball, and Volleyball Players: Systematic Review. Life.

[11] Sánchez-Ruiz R., Gil-Caselles L., García-Naveira A., Arbinaga F., Ruiz-Barquín R., Olmedilla-Zafra A. (2025). Competitive anxiety, sports injury, and playing category in youth soccer players. Children.

[12] Chow A.R.W., Zaneva M., Rashid L., Wheatley C., Coussios C., Hepach R., Bowes L. (2026). Bidirectional relationship between mental health and sports injury in adolescents: A systematic review and meta-analysis. Sports Med..

[13] Dikmen U., Schwab S. (2021). The influence of stress, competitive trait anxiety, and sleep disturbance on injury susceptibility in football. Cent. Eur. J. Sport Sci. Med..

[14] Liberal R., Escudero J.T., Cantallops J., Ponseti J. (2014). Impacto psicológico de las lesiones deportivas en relación al bienestar psicológico y la ansiedad asociada a deportes de competición. Rev. Psicol. Deporte.

[15] Martínez-Estupiñán L.M. (2017). Lesiones deportivas en niños atletas. Estudio de veinte años. MediSur.

[16] Ríos Garit J., Pérez Surita Y. (2021). Factores psicológicos relacionados con las lesiones en deportistas de baloncesto de alto rendimiento en una provincia de Cuba. Rev. Haban. Cienc. Méd..

[17] Ríos Garit J., Pérez Surita Y., Soris Moya Y., Chávez Cárdenas M. (2025). Variables psicológicas predictoras de lesiones en deportistas cubanos de deportes colectivos. An. Acad. Cienc. Cuba.

[18] Olmedilla A., García-Montalvo C., Martínez-Sánchez F. (2006). Factores Psicológicos y Vulnerabilidad a las Lesiones Deportivas: Un Estudio en Futbolistas. Rev. Psicol. Deporte.

[19] Márquez S. (2006). Estrategias de afrontamiento del estrés en el ámbito deportivo: Fundamentos teóricos e instrumentos de evaluación. Int. J. Clin. Health Psychol..

[20] World Medical Association (2013). World medical association declaration of Helsinki: Ethical principles for medical research involving human subjects. JAMA.

[21] Fritz C.O., Morris P.E., Richler J.J. (2012). Effect size estimates: Current use, calculations, and interpretation. J. Exp. Psychol. Gen..

[22] Tomczak M., Tomczak E. (2014). The need to report effect size estimates revisited. An overview of some recommended measures of effect size. Tren. Sports Sci..

[23] Ríos Garit J., Pérez Surita Y., Fuentes Domínguez E., Soris Moya Y., Borges Castellanos R. (2021). Anxiety, and psychological variables of sports performance related to injuries in high-performance sportsmen. Apunt. Sports Med..

[24] Herring S.A., Kibler W.B., Putukian M. (2017). Psychological issues related to illness and injury in athletes and the team physician: A consensus statement—2016 Update. Curr. Sports Med. Rep..

[25] Peñaranda-Moraga M., García-De Frutos J.M., Nadal-Nicolás Y., Cuestas-Calero B.J., Martínez-Rodríguez A., Yáñez-Sepúlveda R., Cortés-Roco G., López-Gil J.F., Muñoz-Villena A.J. (2026). Relationship between psychosocial dimensions of personality and perception of anxiety, with injury history in elite athletes. Front. Sports Act. Living.

[26] Levin S., Tervo T., Ivarsson A., Hägglund M., Stenling A. (2025). Combinations of psychological and physical risk factors for sport injuries in youth floorball players: A latent profile analysis. BMJ. Open Sport Exerc. Med..

[27] Alizadeh M., Pashabadi A., Mohammad S., Shahbazi M. (2012). Injury Occurrence and Psychological Risk Factors in Junior Football Players. World J. Sport Sci..

[28] Núñez A., Kuleva M., Iancheva T., García-Mas A. (2025). Perceived Mental Workload and Psychological Variables in Elite Individual and Team Bulgarian Athletes: An Exploratory Study. Psychol Russ. State Art..

[29] Tutte-Vallarino V., De Los Fayos E.J.G., Serppe M., Malán-Ernst E., Peinado-Portero A., Vilca L.W., Reyes-Bossio M. (2025). Análisis psicométrico del Inventario de Burnout para Atletas-Revisado (IBD-R) en deportistas de alto rendimiento de Uruguay. Retos.

[30] Johnson U., Ivarsson A. (2025). The psychological dimensions of sports injury risk: Models, mechanisms, and interventions. Front. Sports Act. Living.

